# Lactic Acid-Loaded Hydrogels for Post-Episiotomy Wound Healing: Microenvironment Engineering and Regenerative Strategies—A Narrative Review

**DOI:** 10.3390/molecules31071094

**Published:** 2026-03-26

**Authors:** Dragos Brezeanu, Ana-Maria Brezeanu, Vlad Tica

**Affiliations:** 1 6th Department, Faculty of Medicine, Ovidius University of Constanta, 900527 Constanta, Romania; brezeanudragos@gmail.com (D.B.); vtica@eeirh.org (V.T.); 2 County Clinical Emergency Hospital “Sf. Apostol Andrei”, 900500 Constanta, Romania; 3 Romanian Academy of Scientists, 050044 Bucharest, Romania

**Keywords:** lactic acid-loaded hydrogels, episiotomy wound healing, platelet-rich plasma (PRP), microenvironment engineering, regenerative obstetrics

## Abstract

**Background**: Post-episiotomy wound healing remains largely managed through supportive care, despite growing evidence that local biochemical conditions critically influence tissue regeneration. Lactic acid is of particular interest in this context because it is both an endogenous metabolic intermediate and a physiologic component of the vaginal microenvironment, where it contributes to acidic pH maintenance, microbial homeostasis, and mucosal protection. Beyond these local effects, lactate has emerged as a signaling metabolite involved in angiogenesis, immune regulation, and extracellular matrix remodeling, making it a relevant candidate for regenerative wound care. **Methods**: This narrative translational review integrates evidence from molecular biology, biomaterials science, and clinical obstetrics to examine the therapeutic potential of lactic acid-loaded hydrogels for post-episiotomy tissue repair. Literature from PubMed, Scopus, and Web of Science was analyzed to evaluate physicochemical design parameters, lactate-mediated signaling pathways, and available clinical outcomes. **Results**: Lactic acid may function both as a microenvironmental regulator and as a metabolic signal capable of stabilizing hypoxia-inducible factor-1α signaling, enhancing vascular endothelial growth factor expression, modulating macrophage polarization, and influencing fibroblast-mediated extracellular matrix synthesis. Hydrogel matrices provide tunable platforms for controlled lactate release, pH buffering, and mucosal compatibility. Clinical studies suggest improved epithelialization, reduced infection risk, and lower pain scores following topical lactic acid formulations in episiotomy repair. In parallel, platelet-rich plasma provides autologous growth factor enrichment that may complement regenerative signaling pathways. **Conclusions**: Integrating microenvironment stabilization through lactic acid-based hydrogels with biologically active regenerative strategies represents a promising direction for post-episiotomy wound healing. Further controlled trials and standardized biomaterial characterization are required to define optimal therapeutic protocols and confirm long-term clinical benefit.

## 1. Introduction

Obstetric wounds comprise a heterogeneous group of tissue injuries arising during childbirth, most commonly including spontaneous perineal tears, episiotomy-related incisions, and cesarean section wounds [1]. Although these injuries differ in anatomical depth, tissue composition, and surgical context, they share a common clinical importance: their healing quality directly influences postpartum recovery, pain, infection risk, scar formation, sexual function, and overall maternal well-being [2]. In contemporary obstetric care, these wounds are often approached from a procedural or surgical perspective; however, their subsequent repair is also a biologically complex regenerative process shaped by local tissue conditions and systemic host factors [2,3].

Healing of obstetric wounds presents several distinctive challenges [4]. The postpartum environment is characterized by local moisture, microbial exposure, mechanical stress, vascular changes, inflammation, and repeated tissue loading associated with ambulation, urination, defecation, and lochial discharge [5]. These factors may contribute to pain persistence, delayed epithelialization, wound dehiscence, and risk of infection. In addition, patient-related variables such as tissue edema, anemia, nutritional status, metabolic conditions, obesity, smoking, and individual inflammatory response may influence healing trajectories [6]. Compared with many clean surgical wounds, obstetric injuries often develop within dynamic mucosal or highly stressed soft-tissue environments, making their repair especially sensitive to microenvironmental disturbances [4,5,6].

Among these categories, postpartum perineal wounds represent a particularly relevant model for regenerative investigation [7]. They involve a delicate interface between mucosal tissue, resident microbiota, inflammatory signaling, and mechanical vulnerability [8]. Episiotomy, as a controlled and reproducible form of perineal injury, offers a clinically meaningful framework in which healing can be studied with relatively standardized timing, anatomy, and outcome assessment [8,9]. For this reason, although the broader context of obstetric wound healing is acknowledged, the present review focuses primarily on post-episiotomy repair as a translational model for examining how microenvironment-targeted biomaterials may influence tissue regeneration [10].

Wound healing is fundamentally a problem of microenvironmental chemistry [11,12]. Beyond cellular proliferation and tissue approximation, successful regeneration depends on the dynamic orchestration of pH gradients, metabolic intermediates, redox balance, extracellular matrix deposition, and immune signaling within a spatially confined niche [13,14]. Nowhere is this orchestration more delicate than in mucosal tissues, where microbial ecosystems, mechanical stress, and biochemical flux intersect. Post-episiotomy perineal repair represents a paradigmatic example of such a chemically complex healing interface [15,16,17].

Episiotomy creates a controlled but multilayered injury involving stratified squamous epithelium, connective tissue, vascular networks, and muscle fibers [18]. Despite advances in obstetric technique, postoperative management remains largely supportive, relying on hygiene measures and systemic analgesia rather than targeted modulation of the wound microenvironment [19,20]. Yet contemporary insights from immunometabolism and biomaterials science suggest that directing local biochemical conditions may be as critical as mechanical suturing in determining regenerative quality [21].

Lactate has emerged as a central mediator at the interface between metabolism and tissue repair [22]. Long considered a mere end-product of anaerobic glycolysis, lactate is now recognized as a signaling metabolite capable of stabilizing hypoxia-inducible factor-1α (HIF-1α), enhancing vascular endothelial growth factor (VEGF) expression, modulating macrophage polarization, and influencing fibroblast-mediated extracellular matrix synthesis [23,24]. Through these pathways, lactate participates in angiogenesis, inflammatory resolution, and collagen remodeling, core processes of wound healing. Importantly, lactate also contributes to local acidification, thereby exerting antimicrobial effects and influencing epithelial barrier integrity. These dual roles, metabolic signal and pH regulator, position lactic acid as a uniquely multifunctional bioactive molecule in regenerative contexts [25,26]. Lactic acid is particularly relevant to postpartum perineal healing because it is not an exogenous antiseptic conceptually imposed on the wound, but a physiologic metabolite already embedded in the vaginal mucosal ecosystem and increasingly recognized as a regulator of regenerative signaling.

In parallel, the vaginal microenvironment is physiologically characterized by an acidic pH (3.8–4.5), largely maintained by Lactobacillus-derived lactic acid [27]. This acidity is not incidental; it constitutes a chemical defense system that suppresses pathogenic overgrowth and shapes mucosal immunity [28]. Following childbirth, however, tissue disruption and lochia alkalinization transiently alter local pH and microbial equilibrium, potentially affecting inflammatory dynamics and wound repair trajectories [27,29]. Re-establishing controlled acidity during the early postpartum period may therefore have both antimicrobial and immunoregulatory consequences [29,30].

Hydrogel-based biomaterials provide an adaptable platform for precision microenvironment engineering. Through tunable crosslinking density, rheological behavior, osmolarity, and buffering capacity, hydrogels can regulate moisture retention, mechanical protection, and spatiotemporal release of bioactive compounds [31,32]. When functionalized with lactic acid, these systems transcend passive dressing roles and become chemically active scaffolds capable of sustaining localized lactate gradients while preserving mucosal compatibility [33]. The integration of lactic acid within hydrogel matrices allows modulation of proton availability, diffusion kinetics, and interaction with extracellular matrix components—parameters that directly influence cellular behavior and microbial ecology [34].

This convergence of small-molecule bioactivity and polymer-based delivery systems aligns with a broader shift in biomaterials research: from structural substitution toward biochemical instruction [35]. Rather than merely covering the wound, lactic acid-enhanced hydrogels may instruct the perineal niche toward regenerative equilibrium by synchronizing angiogenic signaling, inflammatory resolution, and microbiome stabilization within a chemically optimized scaffold [36,37].

Emerging clinical data suggest improved healing metrics and pain reduction following intravaginal lactic acid formulations after episiotomy, supporting the translational plausibility of this concept [16,38]. However, the molecular mechanisms underpinning these observations, as well as formulation-dependent variables such as pKa-driven buffering behavior, proton diffusion dynamics, and polymer–acid interactions, remain incompletely characterized [39,40].

This narrative review integrates biomaterials science, molecular signaling pathways, and clinical evidence relevant to postpartum perineal tissue repair, with particular emphasis on episiotomy as a reproducible model of obstetric mucosal wound healing. Although cesarean and other obstetric wound categories are briefly referenced for translational context, the primary focus of this review is post-episiotomy healing and the potential role of lactic acid-based hydrogels in modulating its local regenerative microenvironment.

## 2. Post-Episiotomy Wound Healing as a Microenvironmental Challenge

### 2.1. Therapeutic pH Targeting in the Postpartum Perineal Niche

Perineal repair following episiotomy is often approached as a predominantly mechanical event, centered on suture integrity and tissue approximation. However, increasing evidence indicates that biochemical microenvironmental factors play a decisive role in determining the quality and speed of tissue regeneration [41,42]. The vaginal and perineal regions are physiologically characterized by an acidic pH, typically ranging from 3.8 to 4.5, maintained primarily through endogenous lactic acid production by Lactobacillus species [43]. This acidic environment is not incidental; it contributes directly to microbial homeostasis, epithelial barrier preservation, and modulation of local immune responses [44,45].

In the immediate postpartum period, however, this equilibrium may be transiently disrupted [46,47]. Lochial discharge, blood exposure, tissue edema, and mechanical trauma can elevate local pH and alter microbial balance [46,48]. Even modest alkalinization may influence bacterial colonization patterns and inflammatory signaling cascades, potentially affecting the trajectory of wound healing. Within this context, lactic acid-loaded hydrogels are designed not to impose artificial acidification but to restore and stabilize physiologic acidity within a narrow therapeutic range [46].

The acid–base behavior of lactic acid is central to this strategy. With a pKa of approximately 3.86, lactic acid exhibits optimal buffering capacity near physiologic vaginal pH [48]. When incorporated into hydrogel matrices, it can function as a controlled proton donor system capable of counteracting transient alkalinization without producing excessive acidity [49]. The therapeutic aim is therefore dynamic buffering rather than fixed pH reduction. Formulation must carefully regulate lactic acid concentration, degree of ionization, and diffusion kinetics to ensure balanced proton availability. Excessive acid release may provoke epithelial irritation, whereas insufficient buffering may fail to meaningfully influence the wound microenvironment [50,51]. Thus, therapeutic pH targeting represents a chemically precise and clinically sensitive design challenge.

### 2.2. Biocompatibility and Mucosal Tolerance in a Vulnerable Postpartum Setting

The postpartum perineum is a uniquely vulnerable tissue environment [43]. It is highly vascularized, frequently edematous, and subjected to mechanical stress from sitting, ambulation, and defecation [18]. Moreover, mucosal tissues possess distinct permeability and sensitivity profiles compared with keratinized skin [28,51]. Consequently, physicochemical compatibility becomes a central determinant of clinical success.

Osmolarity is a particularly important variable. Hyperosmolar formulations may induce epithelial dehydration, impair cellular function, and delay re-epithelialization [52]. Conversely, excessively hypotonic systems may promote unwanted tissue swelling or dilutional instability [52,53]. Achieving isotonic or near-isotonic balance within lactic acid-containing hydrogels requires careful consideration of total solute concentration, including acid content and excipient contribution [54].

pH stability must also be sustained over time. Fluctuations below physiologic thresholds may lead to discomfort or barrier disruption, whereas upward drift may compromise antimicrobial activity [55,56]. Preservative systems must be selected with caution, as certain antimicrobial additives have demonstrated cytotoxic effects on epithelial cells or may disrupt beneficial vaginal microbiota [56,57]. In this regard, the interplay between lactic acid concentration, buffering behavior, and excipient selection becomes central to mucosal tolerance.

Rheological properties further influence patient experience and therapeutic effectiveness. Formulations that are excessively viscous may impair comfort or spreadability, while low-viscosity systems may fail to maintain adequate residence time. Shear-thinning behavior is advantageous, permitting smooth application under mechanical stress while restoring viscosity at rest. Ultimately, biocompatibility is not an isolated materials property but a multidimensional integration of chemical stability, osmotic balance, rheology, and microbial compatibility.

### 2.3. Controlled Release and Temporal Alignment with Wound Healing Phases

The regenerative potential of lactic acid-enhanced hydrogels is intimately linked to release kinetics. Wound healing progresses through overlapping phases, such as hemostasis, inflammation, proliferation, and remodeling, each characterized by distinct cellular and biochemical demands [38]. Effective microenvironment modulation must therefore align temporally with these dynamic processes.

In the early inflammatory phase, sustained local acidity may help suppress pathogenic overgrowth and support resolution of acute inflammation [38,58]. As healing transitions into the proliferative phase, lactate availability may contribute to angiogenic signaling and fibroblast metabolic activation [58,59]. During remodeling, stabilized extracellular matrix organization may benefit from controlled modulation of local enzymatic activity influenced by pH conditions [60].

Hydrogel matrices enable regulation of these processes through manipulation of crosslink density, mesh size, and polymer–acid interactions. Diffusion of lactate is governed by network architecture and hydration state, allowing gradual release rather than abrupt exposure. Burst release may cause transient discomfort without sustained benefit, while insufficient diffusion may render the formulation clinically ineffective. Thus, optimal hydrogel design requires harmonization between diffusion kinetics and the biological timeline of postpartum tissue repair.

### 2.4. Biological Effects of Controlled Lactate Release on Wound Healing

Beyond its role in pH regulation, controlled lactate release may actively influence several cellular and molecular processes central to tissue repair. Lactate is now recognized not merely as a metabolic by-product, but as a signaling metabolite capable of modulating inflammatory responses, angiogenesis, fibroblast behavior, and extracellular matrix remodeling [11,15,22]. In this context, the therapeutic relevance of lactic acid-loaded hydrogels lies not only in maintaining physiologic acidity but also in enabling sustained local lactate availability during the sequential phases of wound healing [18,24].

One important effect of lactate is its influence on inflammatory regulation. During the early wound-healing phase, excessive or prolonged inflammation may delay epithelial closure and promote tissue breakdown [31,32]. Lactate has been associated with modulation of inflammatory signaling pathways and may contribute to the transition from acute inflammatory activation toward a more reparative microenvironment [35,37]. Lactate has been linked to macrophage polarization patterns associated with tissue repair, favoring phenotypic shifts away from prolonged pro-inflammatory dominance and toward regenerative immune behavior [38,40]. Such effects may be especially relevant in postpartum perineal tissue, where microbial exposure, edema, and mechanical stress may sustain local inflammatory burden [41].

Controlled lactate release may also support angiogenesis. Lactate has been shown to stabilize hypoxia-inducible factor-1α (HIF-1α) under certain tissue conditions, thereby promoting expression of vascular endothelial growth factor (VEGF) and related pro-angiogenic pathways [42,44,45]. In wound repair, these processes facilitate neovascularization, oxygen delivery, nutrient support, and subsequent tissue granulation [46,48]. Sustained rather than abrupt lactate exposure may be particularly important in this context, because angiogenic signaling is temporally coordinated and depends on persistent microenvironmental cues rather than transient biochemical fluctuations [49,50].

Fibroblast activity represents another relevant target. Fibroblasts are central to collagen synthesis, matrix deposition, and wound contraction [51,52]. Lactate has been implicated in fibroblast metabolic activation and in the regulation of genes associated with extracellular matrix production [53,54]. Through these effects, sustained local lactate levels may help support the proliferative phase of healing, during which organized matrix deposition and stromal rebuilding become critical for subsequent scar quality [55]. In addition, pH-sensitive enzymatic activity within the extracellular matrix, including matrix metalloproteinase behavior, may be indirectly influenced by lactate-mediated microenvironmental modulation [56,57].

Re-epithelialization may also benefit from controlled lactate release. Mucosal wound closure requires coordinated epithelial migration and barrier restoration within an environment that remains chemically and microbiologically stable [10,48]. A hydrogel system that provides gradual buffering and sustained lactate delivery may help preserve conditions favorable to epithelial recovery while avoiding abrupt acidification that could irritate damaged tissue [59].

Importantly, the therapeutic value of lactate depends not only on its presence but on its mode of delivery. Sudden or excessive release may lead to short-lived acidification without sustained signaling benefit and may increase the risk of mucosal discomfort [20,60]. By contrast, controlled release from hydrogel matrices may maintain a more stable biochemical niche over time, allowing lactate to function both as a local buffer and as an immunometabolic regulator. This dual mechanism is particularly attractive in postpartum wound healing, where inflammation, microbial ecology, angiogenesis, and matrix remodeling must be coordinated within a vulnerable and mechanically dynamic tissue environment.

Taken together, controlled lactate release should be understood as a microenvironment-guided regenerative strategy rather than a simple acidifying intervention. Its relevance lies in the ability to couple physicochemical stability with sustained biological signaling across the inflammatory, proliferative, and early remodeling phases of wound repair.

### 2.5. Mechanical Compatibility Within a Dynamic Anatomical Region

Beyond chemical considerations, mechanical compatibility is essential for therapeutic persistence. The perineal region is subjected to repeated mechanical loading, stretching, and friction [28,43]. A hydrogel intended for this setting must demonstrate sufficient structural integrity to remain in place without becoming rigid or obstructive.

Elastic compliance compatible with soft tissue deformation is critical. Materials that are too stiff may detach or cause discomfort, whereas overly fluid systems may be displaced rapidly. Mucoadhesion enhances retention, promoting sustained contact between the active formulation and the wound surface. Resistance to dilution by lochial discharge further contributes to therapeutic consistency. Mechanical and physicochemical properties are therefore inseparable determinants of real-world performance.

### 2.6. Microbiome Interaction and Ecological Modulation

Perineal wounds differ fundamentally from cutaneous incisions in that they exist adjacent to a metabolically active mucosal microbiome [61]. Lactic acid-based systems may provide ecological advantages by reinforcing Lactobacillus-dominant flora while discouraging opportunistic pathogens [16,38]. Maintenance of physiologic acidity contributes to microbial balance, which in turn may influence inflammatory signaling and epithelial recovery.

Future translational development should extend beyond macroscopic scar evaluation and incorporate microbiome profiling and inflammatory biomarkers. Understanding how physicochemical modulation affects microbial ecology will provide deeper insight into the mechanisms underlying observed clinical outcomes.

### 2.7. Regulatory Pathways and Translational Implementation

The translational pathway for lactic acid-loaded hydrogels must address regulatory classification, reproducibility, and clinical validation [62]. Depending on formulation complexity and claimed bioactivity, such systems may be categorized as medical devices or combination products [63,64]. Standardization of physicochemical parameters, such as pH, osmolarity, rheology, and release kinetics, is essential for reproducible therapeutic performance [32,63].

Clinical translation should incorporate validated scar scales, pain assessment tools, and long-term functional outcomes, including dyspareunia and tissue elasticity. Stability testing, scalability, and quality control are equally important to ensure consistent safety and efficacy across patient populations.

### 2.8. Clinical Integration Perspective

Viewed through a medical lens, lactic acid-enhanced hydrogels represent an evolution from symptomatic postpartum care toward deliberate microenvironment-targeted intervention. By integrating controlled acidity, sustained lactate availability, mechanical protection, and microbiome compatibility within a biocompatible scaffold, these systems offer a rational framework for modulating early postpartum inflammatory dynamics.

Rather than serving as passive topical agents, they may function as instructive materials that subtly shape the regenerative niche during critical phases of wound repair. Continued interdisciplinary investigation—bridging chemistry, materials science, microbiology, and obstetrics—will determine their ultimate role in advancing evidence-based regenerative strategies for perineal healing.

## 3. Hydrogel Design Principles for Lactic Acid Delivery

### 3.1. Acid–Base Equilibria and Buffering Dynamics in Confined Polymeric Networks

The therapeutic behavior of lactic acid-loaded hydrogels is governed not simply by the intrinsic properties of lactic acid, but by the interplay between acid–base equilibria and the confined architecture of the polymeric network in which the molecule resides [65]. Lactic acid (pKa ≈ 3.86 at 25 °C) exists in dynamic equilibrium between its protonated (HLac) and dissociated (Lac^−^) forms [66]. In free aqueous solution, this equilibrium is defined primarily by bulk pH. Within a hydrogel matrix, however, the situation becomes more complex. Microenvironmental confinement, restricted solvent mobility, ionic strength, and polymer–solute interactions all influence the effective dissociation behavior and proton availability [65,66].

Proton mobility inside a hydrated polymer network is not equivalent to that in bulk water [67]. Network architecture partially constrains diffusion pathways, creating localized pH microgradients that may differ from the external environment [67,68]. In polyelectrolyte systems such as chitosan or polyacrylate-based matrices, Donnan equilibrium effects further modulate ion distribution, altering local proton concentration relative to the surrounding medium. Consequently, buffering capacity becomes a function not only of lactic acid concentration but also of crosslink density, counterion balance, and polymer charge distribution [69,70].

For obstetric applications, this confinement can be advantageous. The physiologic vaginal pH range (approximately 3.8–4.5) lies near lactic acid’s pKa, maximizing buffering efficiency. When properly engineered, the hydrogel functions as a dynamic pH stabilizer, capable of counteracting transient postpartum alkalinization while avoiding excessive acidification that might irritate vulnerable mucosal tissue. Thus, acid–base chemistry within hydrogels is not merely a passive equilibrium, but an actively engineered parameter shaping microenvironmental stability.

### 3.2. Network Architecture, Crosslink Density, and Mesh Size Control

**Figure 1:**
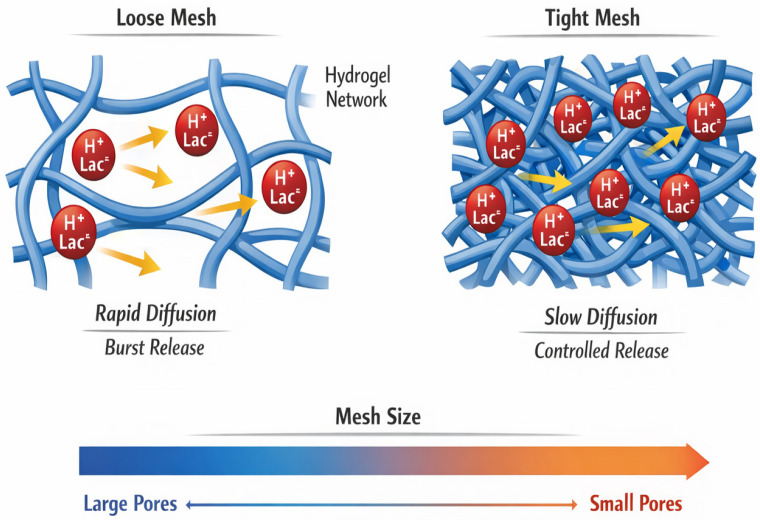
Mesh Size-Dependent Diffusion Model in Functional Lactic Acid-Loaded Hydrogels.

The functional performance of any hydrogel system ultimately reflects its network architecture. Crosslink density (ν_e_) defines the three-dimensional polymer scaffold and directly governs mesh size (ξ), which determines the mobility of small molecules such as lactate [61,62]. In simplified terms, mesh size scales inversely with crosslink density, approximated by ξ ∼ (1/ν_e_) ^(1/3) [62,63]. Although this relationship is idealized, it illustrates a central design principle: tighter networks restrict molecular transport, whereas looser networks facilitate rapid diffusion, as illustrated in Figure 1.

In the context of perineal repair, this architectural control becomes clinically meaningful. Excessively large mesh sizes may permit rapid equilibration of lactic acid, leading to burst release and transient local acidity. Conversely, overly dense networks may impede diffusion to the extent that buffering capacity becomes ineffective. The optimal design therefore lies in a controlled intermediate regime, where diffusion is sufficiently restricted to ensure sustained local modulation but not so constrained as to prevent physiologic interaction with the wound microenvironment.

Material choice further influences this balance. Natural polymers such as hyaluronic acid, gelatin, and alginate offer intrinsic biocompatibility and potential bioactivity, yet may display enzymatic susceptibility and batch variability. Synthetic systems, including PEG-based hydrogels, allow precise control over crosslink density and network homogeneity but often require functional modification to achieve adequate mucoadhesion. Hybrid systems combining natural and synthetic components may provide an optimal compromise between structural reproducibility and biological compatibility.

### 3.3. Swelling Behavior and Osmotic Regulation

**Figure 2:**
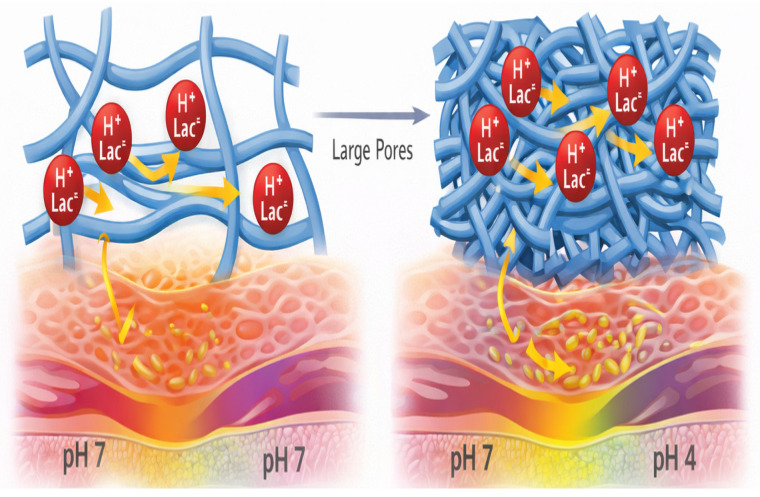
Lactate diffusion and interaction with the perineal wound microenvironment. Schematic representation of loose versus tightly crosslinked hydrogel networks illustrating differences in mesh size (ξ), lactate (Lac^−^/H^+^) diffusion dynamics, and resulting pH gradients at the hydrogel–tissue interface. Larger mesh size permits rapid equilibration and potential burst release, whereas tighter networks restrict molecular mobility, enabling sustained microenvironment stabilization within the postpartum perineal niche.

Hydrogel swelling behavior reflects the dynamic equilibrium between polymer elasticity and osmotic pressure [71]. The equilibrium swelling ratio (Q) directly influences both drug diffusion and mechanical compliance [72]. When exposed to aqueous environments, hydrogels absorb fluid, expanding until elastic restoring forces counterbalance osmotic influx, as illustrated in Figure 2 [73,74,75].

In lactic acid-loaded systems, swelling modulates both concentration gradients and proton mobility. Excessive swelling may dilute local acid concentration and alter release kinetics, while insufficient hydration may restrict diffusion and compromise buffering efficiency [73]. Moreover, osmolarity must remain within physiologically tolerable limits in mucosal applications. Lactic acid contributes to osmotic load, and its incorporation must be balanced with excipients to prevent epithelial dehydration or barrier disruption [74,75].

Thus, swelling behavior is not a secondary characteristic but a primary determinant of both therapeutic stability and mucosal tolerance. Rational formulation requires simultaneous optimization of polymer elasticity, osmotic balance, and active compound concentration to achieve controlled and sustained performance.

### 3.4. Diffusion Modeling and Release Kinetics

The release of lactic acid from hydrogel matrices can be conceptually described using Fick’s second law of diffusion, where the rate of concentration change over time depends on the effective diffusion coefficient (D_eff) within the hydrated network. However, in polymeric systems, diffusion is frequently coupled to polymer relaxation dynamics, leading to anomalous or non-Fickian transport behavior [76,77]. This is particularly relevant in hydrogels with higher crosslink density or dynamic hydrogen bonding interactions.

For postpartum wound applications, release kinetics should align with the temporal sequence of tissue repair. Early inflammatory phases may benefit from stable acid-mediated microbial control, whereas proliferative and remodeling phases require sustained but moderate modulation [38,78]. Therefore, controlled release strategies aim to minimize initial burst phenomena while maintaining gradual equilibration over several days.

Such kinetic control may be achieved through modulation of crosslink density, introduction of ionic interactions between lactate and polymer chains, multilayer or gradient hydrogel architectures, or adjustment of free acid versus lactate salt ratios. These strategies allow fine-tuning of D_eff and overall buffering persistence, translating molecular transport principles into clinically relevant temporal modulation.

This temporal control is biologically relevant because lactate does not act solely as a proton donor, but also as a sustained signaling metabolite. Controlled release may help maintain angiogenic, immunomodulatory, and fibroblast-supportive cues over time, whereas burst release may generate transient acidification without durable regenerative benefit. Therefore, release kinetics should be interpreted not only as a physicochemical property, but also as a determinant of biological instruction within the healing microenvironment.

### 3.5. Rheological and Mechanical Optimization

Beyond chemical functionality, hydrogel performance in perineal repair depends on mechanical compatibility. Storage modulus (G′) and loss modulus (G″) describe elastic and viscous components of material behavior under deformation [79,80]. For mucosal applications, moderate elasticity combined with shear-thinning properties enhances clinical usability. During application, shear stress reduces viscosity, facilitating spreadability; once at rest, structural recovery improves retention and localization. Mucoadhesion arises from hydrogen bonding, electrostatic interactions, and interpenetration between polymer chains and mucin glycoproteins. Effective adhesion ensures that the hydrogel remains localized despite mechanical stress from ambulation, sitting, or lochial flow. Mechanical optimization therefore directly influences residence time, dosing consistency, and therapeutic reproducibility.

### 3.6. Stability, Storage, and Functional Longevity

Long-term functionality requires stability under both storage and physiological conditions. Lactic acid may undergo dissociation shifts with temperature changes, while polymer networks may experience hydrolytic degradation depending on chemical composition. pH drift over time, preservative compatibility, and water activity must all be systematically evaluated [81,82].

Stability testing ensures that buffering performance, diffusion behavior, and rheological properties remain consistent throughout shelf life. Without such validation, even well-designed hydrogel systems may exhibit unpredictable clinical behavior.

In summary, functional lactic acid-loaded hydrogels are not simple topical carriers but chemically and mechanically engineered systems in which acid–base equilibria, polymer network architecture, swelling dynamics, diffusion kinetics, and rheological behavior converge. Their therapeutic effectiveness in obstetric wound repair depends on the precise orchestration of these physicochemical parameters within the biologically sensitive postpartum microenvironment.

### 3.7. Quantitative Considerations: Typical Mesh Size and Diffusion Coefficients in Biomedical Hydrogels

While conceptual discussion of network architecture and diffusion behavior provides a useful design framework, quantitative parameters are essential for translating hydrogel theory into functional biomedical systems [73]. In hydrated polymer networks intended for soft tissue applications, mesh size (ξ) typically falls within the range of approximately 5–100 nm, depending on polymer composition, crosslink density, and degree of swelling. Loosely crosslinked hydrogels based on polyethylene glycol (PEG) or hyaluronic acid often exhibit mesh sizes in the 20–80 nm range, whereas more densely crosslinked or ionically complexed systems may approach lower values, sometimes below 10–20 nm [79,83].

These nanometric structural dimensions are directly relevant to the transport of small molecules such as lactic acid (molecular weight ≈ 90 Da). In bulk aqueous solution at physiological temperature, the diffusion coefficient (D_0_) of small organic acids is typically on the order of 10^−5^ cm^2^·s^−1^ [84]. Within hydrogel matrices, however, effective diffusion coefficients (D_eff) are reduced due to steric hindrance, tortuosity, and polymer–solute interactions. For small molecules in moderately crosslinked hydrogels, D_eff commonly ranges between 10^−6^ and 10^−7^ cm^2^·s^−1^, although tighter networks or highly charged matrices may further decrease transport into the 10^−7^–10^−8^ cm^2^·s^−1^ range [84,85].

This reduction in diffusivity has direct implications for lactic acid-mediated microenvironment stabilization. A decrease of one order of magnitude in D_eff can extend equilibration times from minutes to hours, thereby attenuating burst release and promoting sustained buffering. Excessively low diffusivity may limit effective interaction with the tissue interface, diminishing therapeutic impact. The relationship between mesh size and D_eff is not strictly linear, as polymer relaxation, ionic interactions, and swelling dynamics contribute to anomalous transport behavior [86]. Nevertheless, maintaining mesh sizes within a controlled intermediate regime sufficiently restrictive to modulate transport, yet permissive to ensure physiologically relevant diffusion, appears critical for sustained mucosal microenvironment regulation.

Importantly, swelling state further modifies effective diffusivity. As hydrogels hydrate in situ, increases in water content expand mesh size and enhance molecular mobility. Thus, D_eff in vivo may evolve dynamically over time rather than remaining constant [87]. For obstetric applications, this dynamic adaptation may be advantageous, allowing gradual equilibration during early inflammatory phases while preserving structural integrity throughout proliferative and remodeling stages.

Taken together, quantitative ranges for mesh size and small-molecule diffusion coefficients underscore the feasibility of engineering lactic acid-loaded hydrogels capable of sustained and controllable microenvironment modulation. Incorporating such numerical benchmarks into formulation design enables a more rational alignment between polymer physics and the temporal demands of postpartum tissue repair.

### 3.8. Types of Hydrogels Used in Wound Healing and Tissue Regeneration

Hydrogels used in wound healing and tissue regeneration may be broadly classified into natural, synthetic, hybrid, and stimuli-responsive systems, each offering distinct advantages and limitations depending on the intended biological and translational application [11,13,17]. In regenerative medicine, hydrogel selection is not determined by composition alone, but by how effectively the material integrates hydration control, release kinetics, mechanical compliance, bioadhesion, and tissue compatibility [22,25].

Natural hydrogels, including hyaluronic acid, alginate, gelatin, collagen, and chitosan-based systems, are widely used in wound care because of their intrinsic biocompatibility, high water content, and structural resemblance to extracellular matrix components [28,30,34]. These materials generally support moist healing environments and, in some cases, exhibit favorable bioadhesive or bioactive behavior [36,39]. Chitosan-based hydrogels are particularly attractive in mucosal applications because of their mucoadhesive properties and potential antimicrobial support, which is critical for the vaginal microenvironment [41,43,45]. However, natural hydrogels may also present drawbacks, including batch-to-batch variability, limited mechanical robustness, susceptibility to enzymatic degradation, and less predictable release behavior [48,50].

Synthetic hydrogels, such as polyethylene glycol (PEG)-based, polyacrylate-based, and polyvinyl alcohol-derived systems, provide a high degree of structural tunability and reproducibility [52,55,57]. Their major strengths include adjustable crosslink density, controllable mesh size, predictable swelling profiles, and improved batch consistency. These features make synthetic systems attractive for controlled release applications, including the administration of low-molecular-weight compounds such as lactate or lactic acid, where diffusion depends strictly on the polymer network density [62,64]. Their main limitation is that they often lack intrinsic bioactivity or mucoadhesion and may require blending or surface modification to improve biological interaction with soft tissues [65,67].

Hybrid hydrogels combine natural and synthetic components to balance biocompatibility with physicochemical precision [69,71]. In wound healing and tissue regeneration, hybrid systems are increasingly viewed as advantageous because they can integrate the softness and biointerface compatibility of natural polymers with the reproducibility and release control offered by synthetic matrices [73,74]. For postpartum perineal applications, such a balance may be especially important, given the need for mucosal tolerance, residence time, sustained buffering, and resistance to dilution in a highly dynamic anatomical environment [75,77].

Stimuli-responsive or “smart” hydrogels represent a more recent class of advanced biomaterials [78,80]. These systems can alter swelling, degradation, or release behavior in response to environmental triggers such as pH, temperature, ionic strength, or enzymatic activity [81,82,83,84]. Although still less established in routine clinical obstetric use, they reflect a broader trend in regenerative biomaterials toward adaptive and multifunctional platforms capable of interacting dynamically with the wound microenvironment [85,86,87,88].

Taken together, hydrogel systems for wound healing should be understood not as interchangeable carriers, but as distinct material platforms with specific implications for diffusion behavior, tissue retention, buffering performance, and translational applicability. This distinction becomes especially relevant when considering lactic acid or lactate delivery, where efficacy depends not only on loading capacity but also on controlled microenvironmental modulation.

To provide a more integrated translational comparison, the principal hydrogel platforms relevant to wound healing and potential lactic acid/lactate delivery are summarized in Table 1. The table combines structural class, physicochemical behavior, application setting, and postpartum relevance in a single comparative framework.

As shown in Table 1, no single hydrogel platform can currently be considered definitively superior for lactate delivery in post-episiotomy wound healing. At present, the literature does not support a standardized concentration–response framework for lactic acid delivery in obstetric hydrogel systems, as reported values are heterogeneous and often not directly comparable across studies. Instead, translational suitability appears to depend on the balance between mucosal biocompatibility, mucoadhesion, swelling behavior, structural stability, and release control. Among currently available options, chitosan-containing and hybrid hydrogel systems appear particularly promising because they combine acidic formulation compatibility with improved retention and tunable physicochemical performance.

To further contextualize these findings, Table 2 summarizes individual studies reporting on lactic acid or lactate-containing formulations and hydrogel systems relevant to wound healing, including available physicochemical data, experimental context, and clinical outcomes.

### 3.9. Comparative Performance of Hydrogel Platforms for Lactate Administration

From a translational perspective, the key question is not simply which hydrogel class can incorporate lactic acid, but which material architecture is most capable of sustaining a therapeutically useful lactate microenvironment under the specific constraints of postpartum perineal repair [11,15,20]. As summarized in Table 1, currently available platforms should be interpreted less as competing categories and more as different design strategies, each prioritizing a distinct balance between release control, mucosal tolerance, adhesion, and structural persistence [22,24,28].

For post-episiotomy use, the most critical requirement is not maximal loading capacity, but controlled and biologically tolerable delivery [32,35]. A formulation that releases lactic acid too rapidly may generate transient acidification without durable regenerative benefit, whereas one that retains lactate too tightly may fail to meaningfully influence the tissue interface [38,40]. In this regard, platform performance must be understood as a function of release behavior within a dynamic mucosal environment rather than as a static formulation property [43,45].

Natural hydrogels appear most attractive when tissue compatibility and hydration are the dominant priorities [48,51]. Their softness, high water content, and extracellular matrix-like behavior make them well-suited to vulnerable mucosal surfaces [52,54]. However, these same features may come at the cost of lower mechanical predictability and less precise control over sustained lactate diffusion [55,57].

Synthetic hydrogels occupy the opposite end of the translational spectrum. Their value lies in controllability: mesh size, crosslink density, swelling behavior, and diffusion properties can be engineered with far greater precision than in most biologically derived systems [58,61,63]. Yet in the postpartum perineum, physicochemical precision alone is unlikely to be sufficient [65]. If a material does not adequately adhere to mucosal tissue, accommodate mechanical stress, or maintain patient comfort, theoretical release advantages may not translate into clinical benefit [66,68].

Hybrid systems appear to offer the most plausible compromise between these competing demands [69,71]. By combining the biological compliance and interfacial compatibility of natural polymers with the structural tunability of synthetic networks, they are better positioned to meet the simultaneous requirements of postpartum use [72,74].

Chitosan-containing systems deserve separate attention because they sit at an important translational intersection between natural bioadhesion and acidic formulation compatibility [41,75,76]. Their mucoadhesion, retention, and compatibility with low-pH formulations make them especially attractive for vaginal or perineal application [77,78].

Topical acidic vaginal gels provide an important clinical bridge in this discussion [25,79]. Even when not fully characterized as advanced hydrogel systems, they demonstrate that local lactic acid administration is feasible and tolerable in postpartum care [80,81].

Overall, the comparative interpretation of current hydrogel platforms suggests that future progress will depend less on identifying a universally superior hydrogel class and more on matching formulation design to the biological demands of the postpartum niche. For post-episiotomy repair, the most promising systems are likely to be those that combine moderate crosslink density, stable acidic buffering, mucoadhesion, and sufficient mechanical adaptability to remain functional within a moist and mechanically active environment. On this basis, hybrid and chitosan-containing mucoadhesive hydrogels presently appear to offer the strongest translational rationale, although direct comparative studies remain necessary before any definitive hierarchy can be established.

### 3.10. Key Parameters Controlling Hydrogel Performance

Hydrogel performance for lactic acid administration is governed by a multidimensional set of physicochemical and functional parameters [22,58,82]. First, pH buffering capacity is central because the intended therapeutic effect depends on restoring or maintaining acidity within a narrow physiologic range [18,83]. Second, crosslink density and mesh size regulate lactate mobility and therefore determine whether release is sustained or excessively rapid [60,62].

In addition, rheological properties strongly influence usability and retention [64,84]. Shear-thinning formulations are particularly advantageous in mucosal applications because they facilitate administration while preserving localization at rest [85]. Mucoadhesion is another key determinant, especially in the postpartum setting, where formulation persistence must be maintained despite moisture and discharge [45,77].

Thus, hydrogel efficacy should not be interpreted as a single-variable outcome, but as the integrated result of buffering precision, release kinetics, swelling regulation, rheology, mucoadhesion, osmotic compatibility, and physicochemical stability.

### 3.11. Current Trends in Hydrogel Engineering for Lactate Delivery and Regenerative Obstetrics

Current trends in hydrogel engineering are moving beyond simple passive carriers toward multifunctional and instructive biomaterial platforms [82,86]. One major direction involves smart or stimuli-responsive hydrogels capable of adapting release behavior to pH, temperature, or enzymatic activity [83,85,87].

A second trend is the development of hybrid multifunctional hydrogels that combine synthetic structural control with natural polymer bioactivity [70,74]. A third trend is the integration of bioactive adjuncts, including antimicrobials, probiotics, or autologous biologic products such as platelet-rich plasma, within hydrogel platforms to create dual-action regenerative systems [86,87].

Overall, the field is evolving from conventional dressings toward precision-engineered biomaterials that actively shape the wound microenvironment and interact with regenerative signaling pathways [11,87].

## 4. Clinical Evidence for Lactic Acid in Post-Episiotomy Repair

### 4.1. Evidence Consolidation: The Systematic Review as a Foundational Layer

Translational progress in regenerative medicine requires a structured evolution from dispersed clinical observations to controlled validation. In the context of lactic acid use for episiotomy wound healing, this progression began with systematic evidence consolidation. A recent systematic review synthesizing eight clinical studies evaluating lactic acid in postpartum perineal repair demonstrated consistent benefits across epithelialization time, infection rates, and pain outcomes [16].

Across aggregated datasets, lactic acid-based interventions were associated with approximately 30% acceleration in epithelialization and a 45–50% reduction in infection rates [88,89,90]. Pain scores showed clinically meaningful improvement, with mean reductions of 2–3 points on visual analog scales [88,89]. Meta-analytic evaluation further demonstrated a statistically significant reduction in infection risk (RR = 0.68; 95% CI: 0.52–0.85). Notably, several studies suggested superior outcomes compared with conventional antiseptics such as povidone-iodine or chlorhexidine, agents that may exhibit fibroblast cytotoxicity or epithelial irritation when used repeatedly [91,92,93,94,95].

From a translational perspective, this systematic aggregation established three clinically relevant signals: controlled acidification associated with microbial suppression, improved epithelial regeneration, and enhanced patient-reported comfort [94,95]. However, heterogeneity in formulation type (gel, spray, solution), concentration, and application protocols limited mechanistic clarity and emphasized the need for formulation-specific randomized validation [16].

### 4.2. Randomized Clinical Validation: Intravaginal Lactic Acid Gel as Proof-of-Concept

To address these limitations, a single-center randomized controlled trial evaluated an intravaginal lactic acid gel in 100 postpartum women undergoing mediolateral episiotomy, randomized in a 1:1 allocation [38]. The intervention was administered from postpartum day 1 for seven consecutive days, with assessments performed at day 7 and day 40.

At 40 days postpartum, the intervention group demonstrated significant reductions in scar severity and pain intensity. Effect sizes were moderate to large, with Cohen’s d values of 0.76 for scar healing and 0.83 and 0.79 for VAS and NRS pain scores, respectively. These magnitudes are clinically relevant in obstetric wound care, where topical interventions often yield modest effects [38].

Importantly, hematological parameters, including hemoglobin, hematocrit, leukocyte count, and platelet count, showed no adverse systemic impact. This finding supports the safety of localized application and suggests that the observed clinical benefit is mediated primarily at the tissue microenvironment level rather than through systemic inflammatory modulation.

### 4.3. Mechanistic–Clinical Correlation: A Coherent Biological Narrative

When systematic evidence is integrated with randomized clinical validation, a coherent biologic pattern emerges, as summarized in Table 3.

Notably, early pain differences at day 7 were limited, whereas divergence became pronounced by day 40. This temporal pattern suggests that the benefit of lactic acid is not restricted to immediate antimicrobial effects but extends into proliferative and remodeling phases of wound healing [38,78].

Lactate has been implicated in angiogenesis, fibroblast activation, collagen synthesis, and regulation of matrix metalloproteinases. Therefore, improved scar quality at 40 days may reflect optimized extracellular matrix organization and collagen maturation rather than purely infection control [78]. This aligns with the concept that controlled microenvironment modulation exerts cumulative effects over time, influencing long-term tissue.

### 4.4. Clinical Relevance Beyond Statistical Significance

Effect sizes approaching or exceeding 0.8 represent substantial clinical magnitude in postpartum wound management [38]. Reduced long-term pain may contribute to lower risk of dyspareunia, while improved scar maturation may facilitate functional recovery and maternal comfort. The absence of systemic inflammatory disturbance further supports a localized mucosal mechanism of action, which is particularly relevant in breastfeeding and early postpartum recovery contexts.

In this regard, the postpartum perineum provides a reproducible human model of controlled mucosal regeneration, characterized by predictable timelines and quantifiable endpoints. This setting offers a unique opportunity to evaluate microenvironment-targeted regenerative strategies in a standardized clinical framework [16,38,78].

### 4.5. Translational Implications for Hydrogel-Based Optimization

Although the randomized trial utilized an existing commercial formulation, the findings support further development of purpose-designed lactic acid-optimized hydrogel systems. Refinement of pH stabilization precision, lactate diffusion control, mucoadhesive retention, and targeted release kinetics may enhance therapeutic consistency and maximize regenerative benefit.

The progression from systematic evidence synthesis to randomized clinical validation and mechanistic interpretation outlines a clear translational arc. This trajectory supports continued development of hydrogel-based platforms specifically engineered for postpartum perineal repair, bridging biomaterial chemistry with clinically measurable outcomes.

## 5. Toward Regenerative Obstetrics: Integrating Microenvironment Engineering and Biologic Stimulation

### 5.1. From Passive Healing to Active Regenerative Modulation

In the present review, this regenerative framework is considered primarily through the lens of post-episiotomy perineal repair, while cesarean wound healing is discussed as a related translational comparator rather than as an equally developed review focus.

Obstetric wound healing, whether involving perineal tissue following episiotomy or abdominal layers after cesarean section has traditionally been conceptualized as a predominantly mechanical process [11,12,13]. Surgical approximation is achieved intraoperatively, and the subsequent biological repair is largely left to endogenous regenerative pathways. While this approach is generally effective, it does not actively influence the biochemical and cellular determinants that ultimately shape scar quality, pain trajectories, and long-term functional outcomes [41,42].

**Figure 3:**
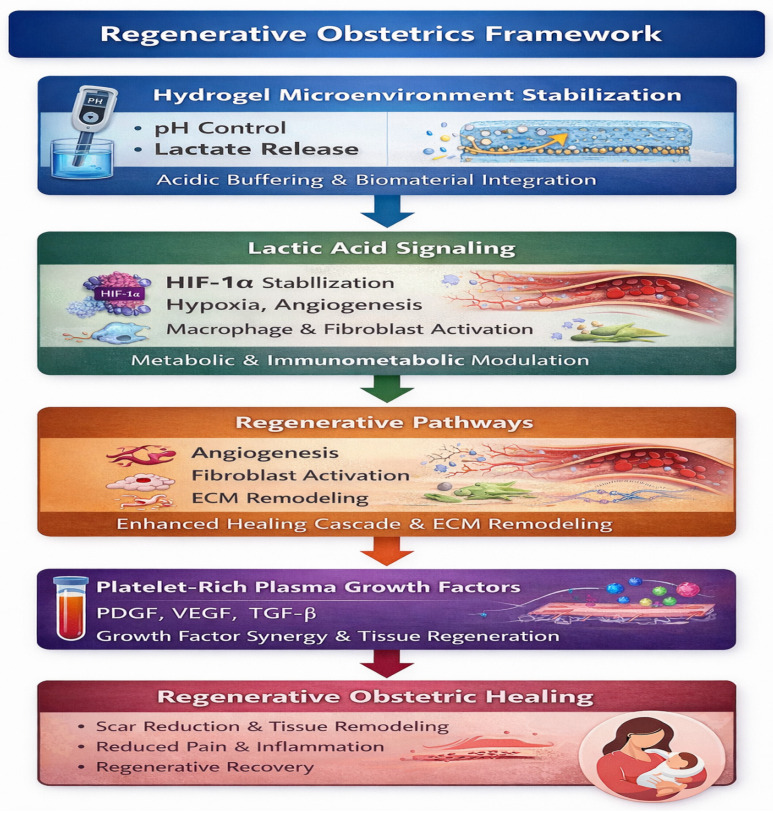
Conceptual framework of regenerative obstetrics.

The integration of physicochemical microenvironment modulation and biologic growth factor stimulation defines an emerging paradigm that may be conceptualized as regenerative obstetrics (Figure 3).

Hydrogel-mediated stabilization of the postpartum wound microenvironment enables controlled lactate signaling, which supports angiogenesis, fibroblast activation, and extracellular matrix remodeling. When combined with platelet-rich plasma-derived growth factors (PDGF, VEGF, TGF-β), these processes converge to promote optimized regenerative healing following obstetric tissue injury.

Emerging clinical evidence suggests that the wound microenvironment is not merely a passive background condition but a modifiable therapeutic target. Parameters such as local pH, inflammatory tone, microbial balance, and growth factor availability critically influence angiogenesis, fibroblast activation, extracellular matrix deposition, and remodeling dynamics. Within this context, two complementary regenerative strategies have gained clinical and translational relevance: chemical microenvironment stabilization through lactic acid-based systems and biologic growth factor enrichment via autologous PRP. Although mechanistically distinct, both approaches converge on shared regenerative checkpoints and offer a foundation for an integrated model of obstetric tissue repair.

### 5.2. Lactic Acid: Microenvironment-Oriented Regeneration

Clinical investigations of intravaginal lactic acid formulations in episiotomy repair have demonstrated significant improvements in scar quality and pain outcomes at 40 days postpartum, with moderate-to-large effect sizes and no evidence of systemic inflammatory disturbance [16,26]. These findings suggest that localized physicochemical modulation may influence tissue repair in a sustained and clinically meaningful manner.

From a mechanistic standpoint, controlled acidification stabilizes microbial ecology within the vaginal–perineal interface, reinforcing conditions favorable to Lactobacillus dominance while suppressing opportunistic pathogens [29]. Beyond antimicrobial effects, lactate functions as an immunometabolic mediator capable of supporting angiogenic signaling, fibroblast metabolic activation, and extracellular matrix synthesis [49,50]. The absence of early dramatic differences at day 7, followed by significant divergence at day 40, supports the hypothesis that lactic acid exerts cumulative effects during proliferative and remodeling phases rather than acting solely as an antiseptic adjunct [38,78].

This strategy may therefore be characterized as microenvironment-first regeneration: it optimizes the chemical niche within which cellular repair processes unfold.

### 5.3. Platelet-Rich Plasma: Growth Factor-Driven Regeneration

In contrast, intraoperative PRP application in cesarean wound repair represents a biologic augmentation strategy centered on concentrated autologous growth factor delivery. Prospective clinical data have demonstrated significant improvement across multiple validated scar scales, including POSAS and REEDA, accompanied by substantial reductions in postoperative pain [96,97]. Correlations between platelet counts and scar outcomes further suggest a dose-dependent regenerative effect.

Mechanistically, PRP introduces a supraphysiologic concentration of growth factors such as platelet-derived growth factor (PDGF), transforming growth factor-beta (TGF-β), VEGF, and epidermal growth factor (EGF) [96,98,99]. These mediators promote fibroblast recruitment, collagen synthesis, angiogenesis, epithelial migration, and modulation of inflammatory cascades. Rather than modifying the physicochemical environment, PRP directly amplifies cellular signaling pathways central to tissue regeneration [100,101].

This approach may therefore be conceptualized as biologic stimulation-first regeneration: it enriches the wound niche with instructive molecular signals that accelerate organized tissue repair.

### 5.4. Convergent Mechanisms: Acid–Growth Factor Synergy

Although developed independently, lactic acid-based microenvironment modulation and PRP-mediated biologic stimulation converge at critical molecular nodes, as shown in Table 4.

Angiogenesis may be supported by lactate-induced HIF-1α stabilization on one axis and VEGF release from PRP on another [89,91]. Fibroblast activity may be enhanced through lactate-driven metabolic priming alongside PDGF-mediated recruitment and activation. Collagen remodeling may be influenced by pH-dependent modulation of matrix metalloproteinases, while TGF-β signaling directs matrix deposition and organization. Inflammatory resolution may be shaped both by controlled acidification and cytokine modulation within PRP preparations [96,97].

Conceptually, lactic acid optimizes the chemical conditions of the regenerative niche, whereas PRP enriches its biological signaling content. These interventions are orthogonal in mechanism yet potentially synergistic in outcome. Together, they define a dual-modulation paradigm in which microenvironmental stability and growth factor-mediated stimulation cooperates to enhance tissue repair.

### 5.5. A Unified Regenerative Model for Obstetric Tissue Repair

Integrating these insights, a unified regenerative framework for obstetric wound healing can be proposed. During the early inflammatory phase, stabilization of local pH and microbial balance may reduce inflammatory persistence, while PRP-derived growth factors initiate angiogenic cascades and fibroblast recruitment. As healing transitions into the proliferative phase, lactate availability may support metabolic activity and angiogenesis, while PDGF and TGF-β promote extracellular matrix deposition. During remodeling, optimized collagen turnover and matrix reorganization may reflect the combined influence of sustained microenvironmental regulation and growth factor-guided maturation.

Notably, both episiotomy and cesarean cohorts demonstrated clinically meaningful improvements by approximately 40 days postpartum, aligning temporally with the transition from proliferation toward early remodeling. This parallel supports the plausibility of shared regenerative mechanisms across distinct obstetric wound types.

### 5.6. Translational Implications: Toward Integrated Regenerative Platforms

The convergence of these strategies raises a natural translational question: could next-generation obstetric regenerative platforms combine intraoperative PRP application with postoperative lactic acid-based hydrogel therapy? Such an approach would address both axes of regeneration biochemical niche optimization and cellular signaling amplification.

Theoretically, PRP could be incorporated into hydrogel matrices, enabling controlled growth factor to release within a stabilized acidic microenvironment. This would represent a transition from isolated adjunctive therapies to bioengineered regenerative protocols. Potential clinical benefits may include reduced long-term dyspareunia, improved scar elasticity, decreased postoperative analgesic requirements, and enhanced overall functional recovery. However, such combination strategies require rigorous physicochemical characterization, safety validation, and controlled clinical evaluation before routine implementation.

### 5.7. Obstetrics as a Translational Model for Regenerative Medicine

Unlike chronic wounds, obstetric incisions occur in generally young, otherwise healthy individuals and follow predictable healing timelines. Surgical technique is standardized, and outcome assessment tools are validated and reproducible. These characteristics render postpartum wound repair an attractive translational model for evaluating microenvironment-targeted biomaterials, autologous biologic therapies, and combination regenerative platforms.

Within this controlled human context, regenerative strategies can be systematically assessed across defined temporal checkpoints, linking molecular mechanisms to clinically meaningful endpoints. As such, obstetric tissue repair may serve not only as a therapeutic target but also as a model system through which biomaterial science and regenerative biology converge in measurable clinical practice.

## 6. Limitations and Future Research Directions

Despite the promising mechanistic rationale and emerging clinical data supporting lactic acid-loaded hydrogels for obstetric wound repair, several limitations warrant careful consideration.

First, the physicochemical discussion presented herein remains largely conceptual and extrapolative. Although principles of acid–base equilibrium, mesh size regulation, and diffusion modeling provide a rational design framework, quantitative in vivo characterization of lactate diffusion coefficients (D_eff), spatial pH gradients, and microenvironmental buffering dynamics within postpartum perineal tissue is still limited. Direct measurement of local pH modulation and lactate concentration profiles in clinical settings remains technically challenging and underexplored.

Second, optimal lactic acid concentration thresholds for maximizing regenerative benefit while avoiding epithelial irritation have not been systematically established. Excessive acidification may disrupt epithelial barrier integrity or alter nociceptive sensitivity, particularly in highly vascularized and inflamed postpartum tissue. The balance between antimicrobial acidity and mucosal tolerance requires further dose–response evaluation.

Third, vaginal microbiome variability represents an important but insufficiently characterized confounding factor. Postpartum microbiota composition differs across individuals and may influence responsiveness to acid-based interventions. Future studies incorporating microbiome profiling and inflammatory biomarkers would provide a more integrated understanding of treatment effects.

Fourth, although preliminary clinical data demonstrate improved scar and pain outcomes, sample sizes remain moderate and the follow-up duration relatively short. Long-term evaluation of dyspareunia, tissue elasticity, and functional recovery beyond 40–60 days postpartum is needed to confirm sustained benefit.

Finally, while combination strategies integrating platelet-rich plasma within hydrogel platforms are conceptually attractive, the stability of growth factors in mildly acidic environments and potential pH-dependent degradation require rigorous biochemical validation prior to clinical translation.

Future research should therefore prioritize quantitative physicochemical characterization, standardized formulation reporting, microbiome-informed endpoints, and adequately powered multicenter randomized trials. Such efforts will determine whether functional lactic acid-loaded hydrogels can be reliably integrated into evidence-based regenerative obstetric practice. Translational development may explore the combined or sequential application of microenvironment-stabilizing biomaterials and biologic augmentation strategies. Such approaches should prioritize safety, reproducibility, and precise physicochemical control to ensure mucosal compatibility and predictable tissue response.

## 7. Conclusions

Obstetric wound healing represents a complex interplay between tissue injury, inflammatory regulation, microbial balance, and extracellular matrix remodeling. The data reviewed here suggest that targeted modulation of the local biochemical environment may influence the quality and trajectory of postpartum tissue repair.

The present review primarily addresses post-episiotomy perineal healing as a clinically relevant model of obstetric wound repair. Therefore, although the mechanistic concepts discussed may hold broader relevance for other obstetric or gynecologic tissue injuries, direct extrapolation to cesarean or more complex obstetric wound categories should be made with caution and requires dedicated investigation.

Lactic acid-enhanced hydrogels offer a chemically rational approach to microenvironment stabilization. By maintaining physiologic acidity and enabling controlled lactate availability, these systems may contribute to antimicrobial balance and support regenerative signaling pathways involved in angiogenesis and fibroblast activation. Complementarily, intraoperative platelet-rich plasma provides an autologous source of growth factors that directly stimulate cellular processes central to wound healing, including neovascularization and collagen deposition.

Although the mechanisms differ, both strategies converge on critical regenerative pathways and have demonstrated encouraging clinical signals in obstetric applications. However, current evidence remains limited by sample size, heterogeneity in formulation and preparation protocols, and relatively short follow-up intervals. Robust multicenter randomized controlled trials, standardized biomaterial characterization, and integration of molecular and microbiological endpoints will be essential to confirm reproducibility and long-term benefit.

In summary, the convergence of biomaterial chemistry and autologous biologic therapies represents a promising but still evolving direction in obstetric wound management. Continued interdisciplinary investigation will determine whether these regenerative strategies can be integrated into evidence-based postpartum care protocols and extended to broader gynecologic surgical contexts.

## Figures and Tables

**Table 1:** Comparative hydrogel platforms relevant to wound healing and potential lactic acid/lactate delivery in post-episiotomy repair.

Hydrogel Type	Representative Materials/Examples	Lactic acid or Lactate Formulation Relevance	Key Physical Properties	Application Context	Main Advantages	Main Limitations	Translational Relevance for Post-Episiotomy Wound Healing
Natural hydrogels	Hyaluronic acid, alginate, gelatin, collagen	Direct lactic acid incorporation is not consistently reported in obstetric studies; relevant as candidate matrices for localized acidic delivery	High water content, strong swelling capacity, soft-tissue compliance, generally limited standalone mechanical strength	Mostly in vitro/in vivo wound-healing and regenerative studies; indirect obstetric relevance	High biocompatibility, moist wound support, ECM-like interaction	Batch variability, enzymatic instability, less predictable release control	Suitable for mucosal repair where hydration and tissue tolerance are priorities
Chitosan-based biological hydrogels	Chitosan hydrogels, acid-containing mucoadhesive gels	Particularly relevant for acidic formulations; specific lactic acid concentration varies by formulation and is not standardized in obstetric literature	Mucoadhesive behavior, moderate swelling, tunable viscosity, moderate mechanical stability, possible antimicrobial-supportive profile	In vitro/in vivo biomaterials studies; indirect translational relevance to postpartum applications	Good mucosal adhesion, compatibility with acidic environments, prolonged residence time	Physicochemical behavior depends strongly on acid type and formulation design	Among the most promising biological platforms for postpartum lactate delivery
Synthetic hydrogels	PEG-based, polyacrylate-based, PVA-derived systems	Strong relevance for controlled lactate release and buffering design, although direct obstetric data remain limited	Tunable crosslink density, predictable mesh size, controlled swelling, improved reproducibility	Mostly experimental drug-delivery and wound-healing systems	High formulation precision, reproducibility, adjustable release kinetics	Limited intrinsic bioactivity, weaker mucoadhesion unless modified	Attractive for engineering sustained and quantifiable lactate delivery
Hybrid hydrogels	Natural–synthetic blends; chitosan–alginate, collagen–synthetic, HA-based composites	Highly relevant for lactate/lactic acid delivery because they can combine retention with release control	Balanced swelling, improved mechanical behavior, adaptable rheology, tunable diffusion	Experimental in vitro/in vivo regenerative systems	Combine biocompatibility with structural tunability, better residence time	Greater formulation complexity and scale-up challenges	Likely the most promising translational direction for post-episiotomy applications
Stimuli-responsive hydrogels	pH-responsive, thermo-responsive, ion-sensitive systems	Potential future platforms for adaptive lactate release; not yet established in obstetric wound care	Dynamic swelling, trigger-responsive release, variable structural stability depending on design	Primarily advanced experimental systems	Smart release behavior, multifunctionality, precision control	Technical complexity, limited clinical validation	High future potential, but still premature for definitive obstetric ranking
Topical acidic vaginal gel systems	Lactic acid-containing vaginal/perineal gels used clinically	Direct clinical relevance for local lactic acid exposure; concentration and release kinetics heterogeneous across studies	Usually softer gel behavior, mucosal spreadability, variable retention and buffering stability	Clinical postpartum/perineal applications	Proof-of-concept clinical evidence, ease of use, tolerability	Often not fully characterized as engineered hydrogels; limited physicochemical reporting	Important bridge between conceptual hydrogel engineering and real postpartum clinical use

**Table 2:** Comparative summary of hydrogel systems and lactic acid formulations relevant to post-episiotomy wound healing.

Study (Ref.)	Hydrogel Type/Composition	Lactic Acid Concentration	Key Physicochemical Properties Reported	Experimental/Clinical Context	Key Outcomes	Relevance to Post-Episiotomy Repair
Brezeanu et al. [38] (Healthcare 2025)	Commercial intravaginal gel (Canesbalance^®^)—carbomer-based acidic gel	Lactic acid-based formulation; exact % not disclosed by manufacturer	Mucosal spreadability; acidic pH buffering; moderate viscosity; gel consistency	RCT, *n* = 100 postpartum women; mediolateral episiotomy; 7-day intravaginal application; outcomes at day 7 and day 40	Significant reduction in scar severity (Cohen’s d = 0.76) and pain (VAS d = 0.83; NRS d = 0.79) at day 40; no adverse systemic effects	Highest-quality direct clinical evidence; proof-of-concept for lactic acid gel in episiotomy healing
Brezeanu et al. [78] (Cureus 2025)	Topical lactic acid gel—not fully characterized as engineered hydrogel	Lactic acid-based; concentration not standardized in report	Gel formulation; mucosal application; pH-buffering intended	Prospective study; post-episiotomy wound healing and sexual quality of life after childbirth	Improved wound healing outcomes; positive impact on sexual QoL postpartum	Supports translational link between lactic acid topical application and functional obstetric recovery
Brezeanu et al. [16] (Healthcare 2025)—systematic review	Various: gels, sprays, solutions containing lactic acid; not exclusively hydrogel-based	Heterogeneous across 8 studies; typical clinical formulations range ~1–3% lactic acid (not standardized)	Variable: pH range ~3.5–4.5; formulation type (gel vs. spray vs. solution); no standardized physicochemical reporting	Systematic review of 8 RCTs/clinical studies; in vivo postpartum perineal repair	~30% faster epithelialization; ~45–50% reduction in infection rates; VAS pain reduction 2–3 points; RR infection 0.68 (95% CI 0.52–0.85)	Foundational evidence layer; establishes clinical signals for lactic acid in perineal healing
Arpa et al. [54] (Gels 2025)	Chitosan hydrogels with organic acids (including lactic acid); mucoadhesive gel systems	Lactic acid used as acidifying/crosslinking agent; specific concentrations tested (formulation-dependent; multiple ratios evaluated)	pH: 3.5–5.0; swelling behavior characterized; viscosity and bioadhesion measured; stability assessed	In vitro physicochemical characterization; mucosal application focus	Lactic acid selection significantly affects bioadhesion, swelling, and stability of chitosan hydrogels; optimal acid type-dependent	Direct relevance: chitosan + lactic acid combination shows strong mucoadhesive profile suitable for postpartum vaginal/perineal use
Lavrentev et al. [77] (Molecules 2023)	Hydrogel matrices (various polymer compositions); diffusion modeling focus	Small molecule diffusion modeled (lactic acid-relevant MW range); no direct LA loading reported	Deff ~10^−6^ to 10^−7^ cm^2^·s^−1^ for small molecules; mesh size ξ ~5–100 nm; diffusion-limited behavior characterized	In vitro/theoretical modeling; drug delivery hydrogel systems	Defines quantitative diffusion parameters for small molecules in hydrogel networks; anomalous/non-Fickian transport identified in dense networks	Provides physicochemical benchmarks for engineering controlled LA release in wound-healing hydrogels
Ho et al. [71] (Molecules 2022)	Multiple hydrogel types: natural (HA, alginate, gelatin, chitosan), synthetic (PEG, PVA), hybrid	Not a lactic acid study; review of hydrogel properties relevant to biomedical use	Swelling ratios, mechanical moduli, mesh size ranges, degradation profiles—class-level data	Comprehensive review; in vitro and in vivo wound healing and tissue regeneration	Establishes comparative physicochemical profiles across hydrogel classes; identifies chitosan and hybrid systems as top candidates for mucosal repair	Provides classification framework directly applied in Table 1 of the present review
Correa et al. [62] (Chem. Rev. 2021)	Translational hydrogels: PEG-based, polyacrylate, natural polymer systems	Not LA-specific; reviews controlled release of small molecules including acids	Crosslink density, mesh size, swelling equilibrium ratio (Q), rheological parameters (G′, G″); mucosal compatibility data	Translational review; in vitro to clinical applications	Defines design parameters for controlled small-molecule delivery; highlights importance of D_eff and network architecture	Underpins hydrogel design framework for LA delivery described in Section 3 of the review
Vigata et al. [64] (Pharmaceutics 2020)	Drug-loaded hydrogels: natural, synthetic, hybrid platforms	Characterization methodology for loaded systems; LA-relevant concentration testing approaches	Release kinetics (Fickian vs. non-Fickian); swelling behavior; pH stability; diffusion coefficients measured	In vitro characterization; drug delivery systems	Standardizes evaluation techniques for hydrogel release systems; burst vs. sustained release profiles characterized	Methodological reference for future lactic acid hydrogel characterization protocols
O’Hanlon et al. [49] (BMC Microbiol. 2019)	Vaginal fluid/acidic formulations; not hydrogel-based	Lactic acid concentrations tested: ~0.5–3% *w*/*v* range; pH range 3.5–5.0	Antimicrobial activity correlated with pH and LA concentration; MIC/MBC-equivalent data for vaginal pathogens	In vitro antimicrobial; vaginal/cervicovaginal microenvironment model	Lactic acid at physiologic concentrations (1–3%) provides significant antimicrobial activity against BV-associated pathogens; pH and LA act synergistically	Establishes therapeutic concentration range for lactic acid in vaginal microenvironment—directly informs hydrogel loading targets
Aldunate et al. [50] (Front. Physiol. 2013)	Vaginal lactic acid—endogenous/formulated solutions; not hydrogel-based	Physiologic lactic acid: ~1–2% in cervicovaginal fluid; L-lactic acid isomer predominant	pH ~3.8–4.5; D- vs. L-lactic acid isomer activity compared; buffer capacity measured	In vitro; cervicovaginal microenvironment; mucosal immunity	L-lactic acid demonstrated superior antimicrobial and immunomodulatory effects vs. D-isomer; pH and isomer type both critical determinants	Informs isomer selection and concentration targets for lactic acid-loaded hydrogels in postpartum perineal applications
Fang et al. [59] (Cell Commun. Signal. 2024)	Not a hydrogel study; lactate signaling review	Tissue lactate concentrations: ~2–20 mM (normoxic); up to ~40 mM (wound/inflammatory context)	Lactate signaling via MCT transporters, HIF-1α stabilization, GPR81; concentration-dependent immunomodulation	In vitro and in vivo; inflammation and wound healing models	Lactate modulates macrophage polarization (M1 → M2 shift), promotes angiogenesis via VEGF, suppresses NF-κB inflammatory axis	Provides molecular rationale for sustained lactate delivery targets from hydrogel systems in post-episiotomy repair
Sim et al. [29] (Int. J. Mol. Sci. 2022)	Acidic wound solutions/topical pH-modulating formulations; not hydrogel-based	pH range tested: 4.0–6.0; lactic acid among acidifying agents evaluated	Tissue pH modulation; in vivo wound pH measurement; correlation with healing rate	In vivo wound healing study; animal models; topical pH intervention	Acidic pH (4.0–5.5) accelerated wound healing vs. neutral pH; enhanced re-epithelialization and reduced bacterial burden	Supports pH-targeting rationale for lactic acid hydrogels; identifies therapeutic pH window relevant to perineal repair

Abbreviations: RCT, randomized controlled trial; LA, lactic acid; HA, hyaluronic acid; PEG, polyethylene glycol; PVA, polyvinyl alcohol; VAS, visual analog scale; NRS, numeric rating scale; QoL, quality of life; BV, bacterial vaginosis; MCT, monocarboxylate transporter; HIF-1α, hypoxia-inducible factor-1α; VEGF, vascular endothelial growth factor; MIC, minimum inhibitory concentration; Deff, effective diffusion coefficient.

**Table 3:** Phase-Specific Regenerative Correlates.

Healing Phase	Observed Clinical Signal	Proposed Lactic Acid Mechanism
Inflammation	Reduced long-term pain	pH-mediated microbial suppression; modulation of TNF-α/IL-6
Proliferation	Improved POSAS scores	Enhanced fibroblast activity; TGF-β1 signaling
Remodeling	Better scar maturation at 40 days	Optimized collagen turnover; ECM reorganization

**Table 4:** Convergent Regenerative Mechanisms of Lactic Acid-Based Microenvironment Modulation and Platelet-Rich Plasma in Obstetric Wound Healing.

Regenerative Target	Lactic Acid Mechanism	PRP Mechanism
Angiogenesis	HIF-1α stabilization	VEGF release
Fibroblast activity	Metabolic signaling	PDGF stimulation
Collagen remodeling	pH-modulated MMP activity	TGF-β signaling
Inflammation control	Acidic antimicrobial effect	Cytokine modulation

## Data Availability

This study is based exclusively on previously published literature. No new experimental or clinical datasets were generated. All referenced data are available in the cited publications.

## Abbreviations

The following abbreviations are used in this manuscript:

PRP	Platelet-rich plasma
HIF-1α	Hypoxia-inducible factor-1α
VEGF	Vascular endothelial growth factor
PEG	Polyethylene glycol
PDGF	Platelet-derived growth factor
TGF-β	Tumour growth factor-beta
EGF	Epidermal growth factor
